# Fire Severity Influences Ecophysiological Responses of *Pinus pinaster* Ait

**DOI:** 10.3389/fpls.2019.00539

**Published:** 2019-04-26

**Authors:** Francesco Niccoli, Assunta Esposito, Simona Altieri, Giovanna Battipaglia

**Affiliations:** Department of Environmental, Biological and Pharmaceutical Sciences and Technologies, Università degli Studi della Campania Luigi Vanvitelli, Caserta, Italy

**Keywords:** fire severity, *Pinus pinaster* (maritime pine), stable isotope of O and C, tree ring, post-fire effects

## Abstract

The effect of fire severity on *Pinus pinaster* growth and ecophysiological responses was evaluated in four burned sites of Vesuvio National Park, Southern Italy. After the wildfire of 2017, when over 1300 hectares of vegetation, mainly *P. pinaster* woods, were destroyed, four sites were selected according to the different degree of fire severity and a multidisciplinary approach based on tree rings, stable isotopes and percentage of crown scorched or consumed was applied. All the sampled trees in the burned sites showed a decrease in tree growth in 2017, in particular in the latewood at high-severity site. The dendrochronology analyses showed that several individuals experienced and endured higher fire severity in the past compared to 2017 fire. Further δ^13^C and δ^18^O underlined the ecophysiological responses and recovery mechanisms of *P. pinaster*, suggesting a drastic reduction of photosynthetic and stomata activity in the year of the fire. Our findings demonstrated that *P. pinaster* growth reduction is strictly linked to the percentage of crown scorch and that even trees with high level of crown scorched could survive. In all the burned sites the high temperatures and the time of exposure to the flames were not sufficient to determine the death of the cambium and all the trees were able to complete the 2017 seasonal wood formation. This data can contribute to define guidelines to managers making post-fire silvicultural operations in pine forest stands in the Mediterranean Basin.

## Introduction

Forest fires are a critical issue in the Mediterranean basin, experiencing increasing frequency and intensity in the last decades (IPCC, 2014).

The impact of fire on trees can cause damage to the canopy, trunk, and root system (Ducrey et al., 1996). The single or combined effect of these damages can reduce the vigor of the plant and trigger a temporary reduction in growth (Battipaglia et al., 2014a) or can lead to tree mortality if the fire is particularly destructive (Brown, 2000). Understanding post-fire responses of trees is a crucial issue in planning forest management actions of burned area in the short term (Bovio et al., 2017) and fire risk reduction at the medium and long term (Battipaglia et al., 2017). It is well-known that the variation in plant responses to fire is linked to the species-specific heat sensitivity (Catry et al., 2010) and to fire regime. Indeed, several plant species are able to tolerate forest fires of medium and low severity, thanks to their adaptive traits that guarantee their survival: very thick bark and needles, deep root system, self-pruning capacity and particular structure of the crown (Brown, 2000; Madrigal et al., 2019). Fire sensitivity is most often studied in pine species due to their wide distribution range (Fernandes et al., 2008; Espinoza et al., 2019). An extensive literature deals with pine post-fire responses and mortality prediction after wildfire or prescribed burnings, mainly in North America (e.g., Peterson and Arbaugh, 1986a; Ryan and Reinhardt, 1988; Stephens and Finney, 2002; Beverly and Martell, 2003; McHugh and Kolb, 2003; Fowler and Sieg, 2004; Kobziar et al., 2006; Sieg et al., 2006; Breece et al., 2008; Hood et al., 2010), but only limited information is related to fire resistance of European pines (see Fernandes et al., 2008). In particular, several papers analyzed post-fire tree mortality or recruitment, while very few concerns the ecophysiological responses of pines (mainly *Pinus halepensis* and *Pinus sylvestris*) after fire (Beghin et al., 2011; De Micco et al., 2013; Battipaglia et al., 2014a,b, 2016; Valor et al., 2018). However, little is known about the post-fire ecophysiological response of *Pinus pinaster* Ait., a widespread and fire-prone species which is also economically and ecologically important in Mediterranean region (Botelho et al., 1998; Vega et al., 2008, 2010; Catry et al., 2010). Previous studies addressed post-fire recruitment (Calvo et al., 2008; Vega et al., 2008, 2010), or post-fire regeneration (Maia et al., 2012) of *P. pinaster*, highlighting contrasting results for this species and its resistance to fire. Indeed, Fernandes et al. (2008) reported a considerable fire resistance of this species due to the bark depth and to the high temperature requested for needle necrosis in comparison to *Pinus pinea*. While Catry et al. (2010), using a mortality model, suggested that *P. pinaster* is more vulnerable to fire than other pines, such as *P. pinea* because of its crown architecture. Finally, Vega et al. (2010), found consistent differences in *P. pinaster* mortality both in relation to site and fire conditions. Thus, it becomes extremely important to better understand ecophysiological responses to fire of this species and its link with fire severity. In this study we used a multidisciplinary approach of dendrochronology, stable isotopes and percentage of crown scorch to assess the effect of a large wildfire, occurred in Italy during July 2017, when over 1300 hectares of vegetation, mainly *P. pinaster* woods, were destroyed and where different degrees of fire damage were recorded (Battipaglia et al., 2017). Dendrochronology allowed, not only, to reconstruct historical fire dynamics (Camarero et al., 2019) in the study area and involving sampled trees, but also to verify the growth related to the fire year 2017. Carbon and oxygen stable isotopes were used to understand the complex ecophysiological processes involved in post-fire responses, linking the possible reduction of carbon assimilation to crown damage or to reduction in stomatal activity (Battipaglia et al., 2014a,b, 2016). Fire severity, considered as a measure of the immediate impact of fire on trees, was evaluated in terms of the degree of crown scorch, cambium or root damage and height of burn on the trunk (Van Wagner, 1973; Moreno and Oechel, 1989; Lentile et al., 2006; Keeley, 2009).

This research aims to improve our understanding of the wildfire effects on *P. pinaster*, with particular focus on the strategies and mechanisms that this species is able to adopt to face the passage of fire.

We aim, not only, to verify the link between fire severity and *P. pinaster* ecophysiological responses, but also to understand the possibility of its survival in the short and long term. We hypothesize a tight relation between fire severity, in particular crown scorch, and tree damage in the short term, with needle defoliation triggering growth reduction and decrease in photosynthetic activity. Further, we hypothesize that stable isotopes can help assessing the ecophysiological processes activated by fire and can contribute to determine possible species survival. In particular, carbon stable isotope can be considered a proxy of changes in the gas exchange processes and can be used to estimate the ratio between photosynthetic activity (A) and stomatal conductance (gs) (Farquhar et al., 1982). Whereas δ^18^O can help elucidating the independent effect of A and gs on δ^13^C (Scheidegger et al., 2000). Indeed, high fire severity can damage the crown, reducing leaf surface and altering the plant’s photosynthetic rate and efficiency (Beghin et al., 2011).

This research appeared extremely important from a forest management point of view, since the right assessment of actual tree damage and a better prediction of post-fire tree survival could avoid cutting down a scorched tree that could not be dead. Indeed harvesting trees, when is not necessary, could alter the post-fire germination and, generally, the carbon cycle and the ecosystem biodiversity.

## Materials and Methods

### Study Area

**FIGURE 1:**
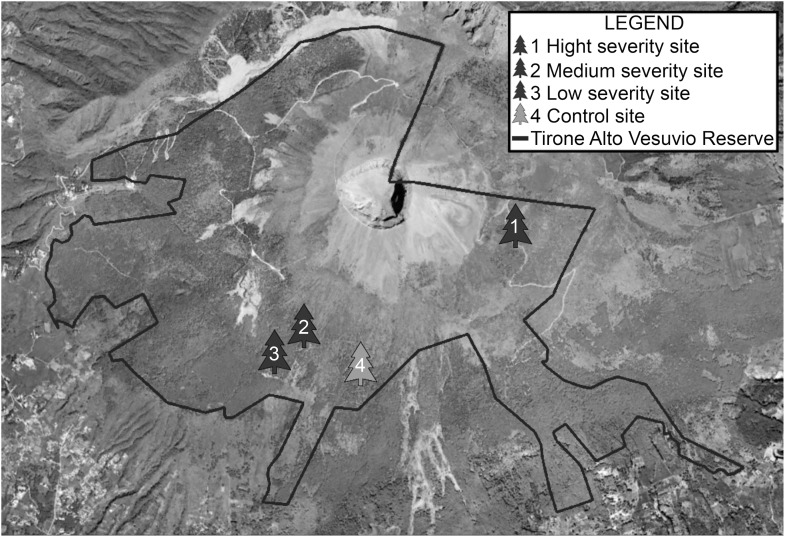
Sampling sites in the Tirone Alto Vesuvio Nature Reserve of the Vesuvio National Park (Italy).

The study area is the “Tirone Alto Vesuvio Nature Reserve” within the Vesuvio National Park (Figure 1), which covers a total area of 8482 hectares and suffers a strong anthropic pressure: almost 400000 inhabitants live in the 13 municipalities around the protected area. The Park, is characterized by a large plantation forest: while the Northern slope is covered by tall deciduous trees, the Southern side presents a large plantation dominated by *P. pinaster* Aiton and *P. pinea L*, with the presence of *Pinus halepensis* Mill, *Pinus nigra* Arnold (Picariello et al., 2000) and scattered patches of typical Mediterranean macchia vegetation. The study area shows the typical Mediterranean climate, with hot and dry summers and rainy winters with mild temperatures. In the last 40 years the average temperatures have gradually increased from 14 to 16°C and the rainfall has been very erratic: some years (1980, 1984, 1996, 2005, 2009, 2010) have been characterized by heavy rains, others have been extremely dry (1977, 1999, 2003, 2012, 2016, 2017) (The Royal Netherlands Meteorological Institute, KNMI database Climate Explorer^1^, Trouet and Van Oldenborgh, 2013 and Supplementary Figure S1, data acquired on January 2018). The frequent drought seasons, the presence of strong winds and particularly flammable vegetation, associated with a strong anthropic pressure, make the area particularly vulnerable to forest fires (Sibilio et al., 2002).

To evaluate the effects induced by the fire of July 2017 on *P. pinaster* plantation, four sampling sites (around 1 ha) were chosen within the Tirone Alto Vesuvio Nature Reserve, from areas burned with different fire severity (Table 1). Fire severity was evaluated based on parameters such as: presence and height of scorch on the trunk, presence of fire scars on the trunk, percentage of consumed crown and presence of damage to the roots related to 2017 fire.

**Table 1:** Sites coordinates, historical fire events, tree average diameter at breast height (DBH), tree height and severity parameters (percentage of crown reduction, height of scorch on the trunk) of 2017 wildfire.

					2017 fire severity	
Site	Coordinates	Fire events	*P. pinaster* DBH	*P. pinaster* height	% Mean crown reduction	Height of scorch on the trunk
HSS	Latitude 40° 48′ 51.12″ N Longitude 14° 26′ 20.12″ E	2017	28.2 ± 5.9 cm	13.1 ± 0,17 m	70	<4 m
MSS	Latitude 40° 48′ 45.35 ″N Longitude 14° 24′ 49.65″ E	1993, 2007, 2015, 2017	34.4 ± 4.3 cm	17 ± 0,24 m	10	3 m
LSS	Latitude 40° 48′ 43.00″ N Longitude 14° 24′ 44.64″ E	1993, 2014, 2016, 2017	34.5 ± 3.3 cm	20 ± 0,16 m	2	2.5 m
CS	Latitude 40° 48′ 35.80″ N Longitude 14° 24′ 52.70″ E	-	26.2 ± 6.1 cm	14.6 ± 0,24 m	–	–

*HSS, high severity site; MSS, medium severity site; LSS, low severity site; CS, control site.*

Fire severity was high in the high severity site (HSS), in which the individuals of *P. pinaster* showed important damages to the canopy (70% crown reduction) and scorch on the trunk (over four meters). The severity was medium in the medium severity site (MSS), where the plants showed partial damage of crown (10%) and a scorch on the trunk that not exceed three meters. In addition, low severity was estimated in the low severity site (LSS), where individuals presented mild damage to the needles (average reduction of 2%) and scorch of the trunk of two and a half meters. Finally, individuals were sampled in control site (CS): an area situated into the Nature Reserve but where individuals did not show any scorch or damage to the trunk roots or to the crown trees, since they were not affected by the fire event of 2017.

Evidences of past fire events related to the four study sites were collected using the official fire reports, from 1988 to 2016, of the “Carabinieri Forestali per la Biodiversità (UTCB) of Caserta,” a governmental agency, acting since 1974, as a park ranger force.

In addition to the large fire of the 2017, two surface fires, in 2015 and 2007, occurred in the MSS. Further in August 1993, an important wildfire (more than 240 he was burned) occurred on the Southern slope of Vesuvio, including the MSS and LSS sites. This information was, subsequently, related to dendrochronological (especially to fire scars) and isotope data. Finally, the LSS site was subjected to prescribed fires in 2014 and 2016, promoted by the Campania Region (Battipaglia et al., 2017).

### Sampling and Processing of Tree Cores

In December 2017, for each study site, 15 dominant trees (trees with crowns extending above the general level of the main canopy and receiving full light from above and partly from the sides- Adams et al., 1994) were sampled with a 5 mm increment borer (Haglöfs, Långsele, Sweden), and from each individual two cores were collected, at 1.3 m above ground, at east and west direction.

The total of 120 collected cores were fixed on specific wooden supports and, subsequently, subjected to a sanding process, to facilitate tree-ring identification. The measurement of the tree-ring growth was carried out through the LINTAB system: a stereo-microscope connected to a computer, in which a specific software called TSAP-Win is installed and allows to elaborate a series of representative curves of each individual plant growth trend. After measuring each sample, first a visual comparison, then a statistical synchronization of the curves, known as cross-dating, was performed using the Gleichaeufigkeit index (GLK), which evaluates the correlation between the different series (Eckstein and Bauch, 1969).

Successively, the data were statistically analyzed using the COFECHA software, which allowed checking the quality of cross-dating, providing indications on the number of years to add or subtract to the chronologies.

Finally, the elementary chronologies have been standardized using ARSTAN (Cook and Holmes, 1986). Standard chronologies were detrended using the smoothing spline function, with a 40-year step for the chronologies of MSS site and a 10-year step for those relating to the HSS, LSS, and CS sites. The mean chronology was calculated through an arithmetic mean, while the stabilize variance was determined through the Keith Briffa rbar-weighted method. Finally, a different running bar was used for each site, allowing to maximize the expressed population signal (EPS).

### Sample Pretreatment and Isotopic Analysis

For each site five cores were selected for isotope analysis using the Gleichaeufigkeit (GLK) which evaluates the correlation between the different series (Eckstein and Bauch, 1969). The five selected cores presented the best cross-dating (GLK > 0.70) with the corresponding average chronology. The annual rings of the last 30 years (1988–2017) were manually removed and divided into late-wood and early-wood. Only for the CS site, with the youngest trees, the isotopic analyzes were performed across a shorter time period (1994–2017). Further analyzes were performed on an individual of the MSS who had an obvious fire scar (see Supplementary Figure S2) belonging to a past fire event.

Subsequently, the samples were milled using a pulverizing mill and were weighed precisely and encapsulated in a tin or silver capsules for the carbon or oxygen isotopic measurements, respectively.

The carbon and oxygen stable isotope composition was measured by continuous-flow isotope ratio mass spectrometry (Delta V Advantage, Thermo Scientific) at IRMS lab of University of Campania. The δ^13^C series were corrected for the fossil fuel combustion effect (Francey et al., 1999).

### Climate Analysis

Climate data of minimum, maximum, and mean monthly temperature and total monthly precipitation for the period 1975–2017 were downloaded from the CRU TS3.23 gridded dataset at 0.5° resolution data (Harris et al., 2014) since Zalloni et al. (2018) demonstrated for the same area the high significant correlation with local meteo stations. A Pearson’s linear correlation function analysis (*P* < 0.05), performed using Excel, was implemented between climate data and tree-ring width and isotope data. Temperature and precipitation data were seasonally grouped from December of the previous year to February of the next year, in order to cover all the season which could influence tree-ring growth in Mediterranean species (Cherubini et al., 2003; Balzano et al., 2018). Those relationships help clarify the role of climate, namely temperature and precipitation, on past tree growth and to understand how much it influenced tree growth in CS site, not affected by fire.

## Results

### Dendrochronological Data

**FIGURE 2:**
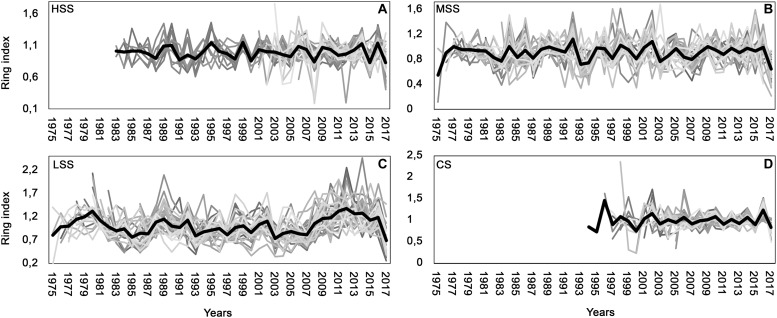
Individual standardized chronologies in gray and average chronologies in black for **(A)** high severity site (HSS), **(B)** medium severity site (MSS), **(C)** low severity site (LSS), **(D)** control site (CS).

The standardized average chronologies (Figure 2) of the individual study sites showed a discrete synchronization: of the 43 years analyzed, the growth trend was common for 25 years. The good synchronization of the chronologies of the study areas is confirmed by the EPS value > 0.85 (Table 2). Also, the average sensitivity values of the four sites are very similar (always < 0.25): the sampled trees can be considered compliant, even if it is necessary to underline that the plants of the control area have a particularly high MS.

The HSS chronologies (Figure 2A), showed a good consistency in growth trend (EPS = 0.9; GLK > 60; MS = 0.12). In all individual chronologies, 1999 presented a simultaneous growth increase while a drastic growth reduction was observed in 2008, 2012, and 2017.

The MSS and LSS sites (Figure 2B,C) are very similar both in terms of age (mean age MSS = 38 years, mean age LSS = 37 years) and in terms of growth. Some years present a common increase of growth (e.s., in 1989, 1992, 1998, 2002) or a common decrease of growth (es., in 1993, 1997, 2000, 2003). In particular, a significant difference in growth between sites was observed in the period 2014–2016 (Figure 2C), in which there was an increase in tree growth in the individuals of the LSS site.

Finally, the CS (Figure 2D) presented the youngest trees (average age = 16 years) with positive growth peaks during 1999, 2002, 2006, 2010, 2014, 2016 and negative growth in 2000, 2003, 2005, 2007, 2011, 2013, 2015.

**FIGURE 3:**
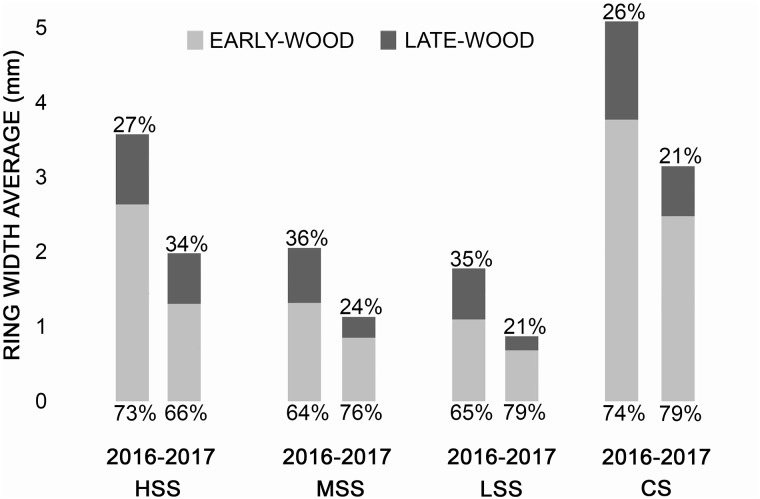
Annual ring width of the 2016 and 2017 reported in each site. In gray the early-wood and in black the late-wood with the percentage of growth, respectively.

The tree-ring width showed that in 2017 all the sampled trees recorded a decrease of growth both in early-wood and latewood, in comparison to the previous year 2016 (Figure 3). In particular, in comparison to 2016, at HSS the total decrease in growth was 55,6% (early-wood decrease of 49,8%, late-wood 72%) while at the MSS of 55,7% (early-wood decrease of 65%, late-wood 38,5%) and LSS of 49,5% (early-wood decrease of 63,2%, late-wood 27,5). Finally, at CS the total decrease of the growth was 62,4% (early-wood of 66,4%, late-wood 50,8%).

**Table 2:** Statistical parameters of the indexed average chronologies of the four areas.

	HSS	MSS	LSS	CS
Mean TRW index	0.994	0.987	1.010	0.993
Standard deviation	0.092	0.099	0.175	0.156
EPS	0.905	0.908	0.946	0.889
MS	0.121	0.116	0.126	0.223

*TRW, tree ring width; EPS, expressed population signal; MS, mean sensitivity.*

**FIGURE 4:**
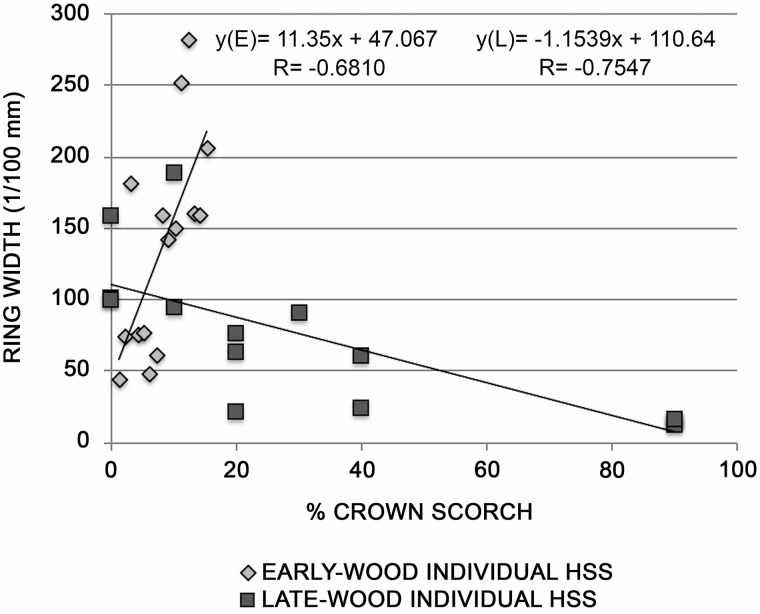
Relationship between tree-ring widths of early-wood (E) and late-wood (L) of HSS site and the % of scorched crown.

The tree-ring widths of 2017 of each individual of the LSS, MSS (see Supplementary Figure S3) and HSS sites (Figure 4), were related to the percentage of destroyed crown. In the HSS a strong negative correlation was found, suggesting that the decrease in growth is directly proportional to the amount of needles destroyed by the fire: the plants that have a 90% crown reduction, showed a strong decrease in growth. In particular, relating the percentage of scorched crown with the early-wood and late-wood ring-width, we observed that the highest negative correlation was found with late-wood. Indeed, the 2017 fire occurred in July, the period when the late-wood is normally formed.

### Carbon Isotopes

**FIGURE 5:**
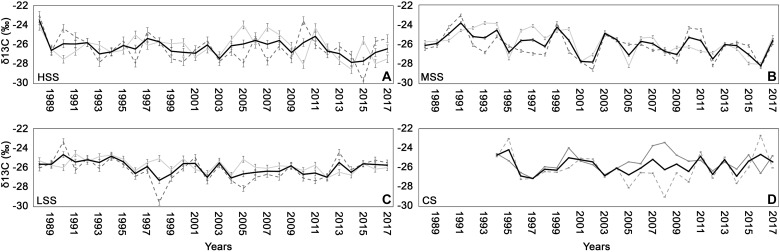
δ^13^C chronologies (± standard deviation) for early-wood (gray line), late-wood (dashed gray line), and for the whole wood (black line) for **(A)** high severity site (HSS), **(B)** medium severity site (MSS), **(C)** low severity site (LSS), and **(D)** control site (CS).

The HSS presented a δ^13^C mean value, recorded between 1983 and 2017, of −26.3 ± 0,4‰ (Figure 5A), at MSS and LSS the average δ^13^C, measured between 1975 and 2017, was −26 ± 0,14‰ for MSS and −26.01 ± 0,3 for LSS (Figure 5B,C), finally in the CS the C isotope values, for the period 1994 and 2017, was −25.7 ± 0,05‰ (Figure 5D). The δ^13^C site-chronologies showed different trends with only MSS and LSS presenting a high correlation (*r* = 0.26, *P* < 0.05). However, some years influenced the δ^13^C values of the four sites in the same way: negatively during 1993, 2012, 2014, positively during 1994, 2006.

While the 1993 fire drastically lowered the δ^13^C in all sites, the fires of 2007 and 2015 caused the decrease only in MSS site.

The different trends are also evident for δ^13^C measured in the early-wood and in the late-wood of the four sites (Figure 5) with the exception of particular years (2000, 2001, 2012, 2014, 2016, and 2017 for early-wood and, 1990, 1993, 1994, 1996, 2005, 2006, 2013 for late-wood), in which the isotopic values showed a similar response (relationships rise or fall at the same time). During the 2017 the δ^13^C values recorded in whole wood and particularly in late-wood resulted to increase at the HSS and MSS sites, compared to the previous year. At the LSS the δ^13^C of whole wood was almost unchanged in 2017 compared to 2016, with slightly decrease in late-wood while at CS we observed a decrease in δ^13^C.

### Oxygen Isotopes

**FIGURE 6:**
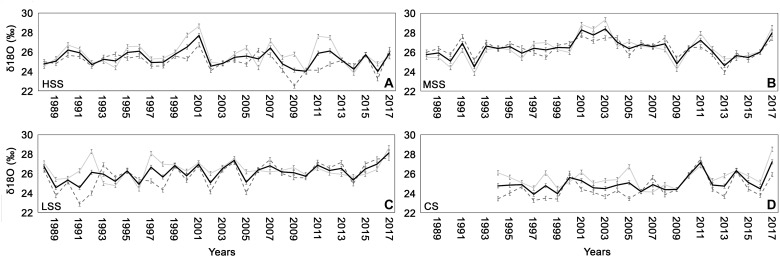
δ^18^O chronologies (± standard deviation) for early-wood (gray line), late-wood (dashed gray line), and for the whole wood (black line) for **(A)** high severity site (HSS), **(B)** medium severity site (MSS), **(C)** low severity site (LSS), and **(D)** control site (CS).

The δ^18^O values of the four sites varied in a range between 23.8 and 28.39‰ for the study period (Figure 6). The four chronologies showed a low synchronization among each other with only few years presenting the same trend, i.e., 2001, 2002, 2011, 2012, and 2017 both in whole wood and in each wood compartment (early-wood and late-wood). In particular δ^18^O of 2017 strongly increased in all the sites.

### Relationships Among Climate, Growth, and Isotopes

The mean temperature, especially during the summer (JJA = June, July, August), was negatively correlated with the annual growth of the plants of all the four sites (Table 3). While the mean temperature of spring (MAM = March, April, and May) and autumn (SO = September, October) led to an increase of δ^13^C in the sampled individuals. The mean temperature of spring (MAM) and of summer period (JJA) triggered the increase of δ^18^O. A high positive correlation was found between total annual rainfall and tree growth of HSS, LSS and CS sites. While negative correlation resulted between total precipitation and the late-wood δ^13^C of LSS site.

**Table 3:** Correlations between whole width (TRW), early-wood (TRW EW), late-wood (TRW LW), δ^13^C of whole wood (δ^13^C TOT), δ^13^C C of early-wood (δ^13^C EW), δ^13^C of late-wood (δ^13^C LW), δ^18^O of whole wood (δ^18^O TOT), d δ^18^O, of early-wood (δ^18^O EW) and δ^18^O of late-wood (δ^18^O LW) of the four sites, with monthly mean temperature (Tmean) (MAM, March, April, and May; JJA, June, July, and August; SO, September and October; ND, November and December) and total annual precipitation (Ptot).

Climate factor	Variable	Month	*R*	Study area
Tmedium	TRW EW, TRW TOT, δ13C TOT	MAM	−0.34^∗^	HSS
Tmedium	TRW EW	JJA	−0.54^∗∗∗^	HSS
Tmedium	TRW TOT	JJA	−0.52^∗∗^	HSS
Tmedium	TRW EW	ND	−0.46^∗∗∗^	HSS
Tmedium	TRW TOT, δ13C LW	ND	−0.41^∗^	HSS
Tmedium	δ13C TOT	ND	−0.52^∗∗^	HSS
Ptot	TRW LW	JA-DE	0.47^∗∗^	HSS
Ptot	TRW TOT	JA-DE	0.35^∗^	HSS
Tmedium	TRW EW, TRW LW, TRW TOT	MAMA	−0.60^∗^	MSS
Tmedium	δ13C LW, δ13C TOT	MAM	−0.37^∗^	MSS
Tmedium	TRW EW, TRW LW, TRW TOT	JJA	−0.75^∗∗∗^	MSS
Tmedium	δ18O EW	JJA	0.41^∗^	MSS
Tmedium	TRW EW, TRW TOT	SO	−0.63^∗∗^	MSS
Tmedium	TRW LW	SO	−0.43^∗∗^	MSS
Tmedium	TRW EW, TRW TOT	ND	−0.63^∗∗^	MSS
Tmedium	TRW LW	ND	−0.46^∗∗^	MSS
Ptot	RAW LW	JA-DE	0.31^∗^	MSS
Tmedium	TRW EW, TRW LW, TRW TOT	MAM	−0.52^∗∗∗^	LSS
Tmedium	δ13C EW	MAM	−0.30^∗^	LSS
Tmedium	δ18O LW	MAM	0.36^∗^	LSS
Tmedium	TRW EW, TRW LW, RAW TOT	JJA	−0.79^∗∗∗^	LSS
Tmedium	δ18O LW, δ18O TOT	JJA	0.44^∗∗^	LSS
Tmedium	TRW EW, TRW LW, TRW TOT	SO	−0.55^∗∗∗^	LSS
Tmedium	δ18O LW, δ18O TOT	SO	0.32^∗^	LSS
Tmedium	TRW EW, TRW LW, TRW TOT	ND	−0.62^∗∗∗^	LSS
Tmedium	δ13C EW	ND	−0.32^∗^	LSS
Ptot	δ13C LW	GE-DE	−0.31^∗^	LSS
Tmedium	δ13C EW	MAM	0.45^∗^	CS
Tmedium	TRW EW, TRW LW	JJA	−0.43^∗^	CS
Ptot	TRW EW	GE-DE	0.64^∗∗∗^	CS
Ptot	TRW TOT	GE-DE	0.60^∗∗^	CS

*R indicates the correlation coefficient of Pearson, with the corresponding significance (^∗^P < 0.05, ^∗∗^P < 0.01; ^∗∗∗^P < 0.001) calculated according to Student’s t-test.*

### Isotopic Response of an Individual With 1993 Fire Scar

**FIGURE 7:**
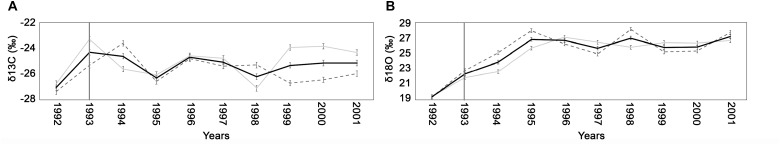
Trend of the δ^13^C **(A)** and the δ^18^O **(B)** ( ± standard deviation) for early-wood (gray line), late-wood (dashed gray line), and for the whole wood (black line) recorded in the individual with a 1993 fire scar.

The analysis of stable isotopes, focused in the period 1992–2001, showed how, due to the fire event of 1993, the δ^13^C and the δ^18^O increased sharply (Figure 7A,B). The carbon isotopic composition spanned from −28.24‰ of the spring ‘92 to −23.51‰ of summer ‘93, while oxygen ranged from 20.33‰ of 1992 to 23.39‰ of summer ‘93. After 1993, the δ^13^C values were stabilized while the δ^18^O continued to increase, reaching, in 1995, a value of 28‰. The normal trend was recovered only in 1998.

## Discussion

### Fire Effect on Tree Growth

*Pinus pinaster* trees sampled in the three burned sites (HSS, MSS, and LSS) showed a decrease in tree growth in 2017, in particular in the late-wood at HSS. Indeed, the fire event occurred in July, when the late-wood is formed in several woody species growing on Vesuvio area (Balzano et al., 2018). Reduction in growth has been described as a short term effect of fire in many species and across different ecosystems (e.g., Hoffmann and Solbrig, 2003; Werner, 2005; Werner et al., 2006; Goldammer, 2007; Murphy et al., 2010; De Micco et al., 2013; Battipaglia et al., 2014b) and it has been interpreted as an abrupt narrowing of growth rings after fire (Schweingruber, 1993; Stahlea et al., 1999) mainly due to cambium or crown damage (Pausas et al., 2003). When the tree is seriously damaged, the formation of new cell can be interrupted in the injured sector of the trunk or branch and fire scars can be formed (De Micco et al., 2014). No fire scars have been observed after the 2017 fire in all the burned sites, further all the sampled trees, including the one growing in the HSS, were able to complete the 2017 seasonal wood formation.

For *P. pinaster*, the bark is considered the main adaptive trait in response to forest fires (Ryan et al., 1994). As for *P. pinea*, the bark of the analyzed species is laminated, a peculiarity that allows to the outer layers of the trunk a gradual exfoliation during combustion, which contributes not only to the dispersion of heat, but also to an increase in the time necessary for killing the vascular cambium (Peterson and Ryan, 1986b; Rego and Rigolot, 1990; Battipaglia et al., 2016). The bark thickness is strongly dependent on tree age (Fernandes and Rigolot, 2007), indeed its diameter doubles when the plant reaches 10 years of age. The maritime pine (*P. pinaster*) growing at the study sites presented a DBH (diameter at breast height) > 20 cm, with a fairly thick bark (between 1.5 and 4 cm), which guarantees a good defense against fire (Burrows et al., 2000). This could explain why, even if the sampled individuals in the HSS, MSS, LSS presented a considerable bole char height, reaching the 4 m in the HSS, no fire scar were formed.

For the majority of conifers crown volume damaged is considered the most important factor determining mortality (Sieg et al., 2006). Previous studies (Freitas, 1995) demonstrates that the needles of the *P. pinaster* are less susceptible to thermal trauma than those of *Pinus halepensis* and *P. pinea*. In fact, maritime pines are able to survive at temperatures between 55 and 65°C, for a time of exposure to the flame of 60 s. Also, the apical buds show a remarkable resistance to combustion: the protection offered by the surrounding long needles shield the gem from the connective heat of the flame (Michaletz and Johnson, 2006). Therefore, it seems evident that the burning of the needle in *P. pinaster* is not always a symptom of the destruction of the crown: even in the case of complete defoliation, the plant has been found able to survive (De Ronde, 1983). Experimental prescribed burning, carried out on the maritime pine by in McCormick (1976), have allowed to report the damage to the foliage with the respective growth rate, showing that, in adult individuals, a slight damage of the crown does not lead to a drastic decline in growth. On the contrary, when the crown damage exceeds 25%, it is possible to find a substantial decrease in growth. Our findings are in agreement with McCormick’s studies: the plants of the MSS and LSS that showed a reduction of the crown of 10% did not show particular growth decreases, while the individuals sampled at HSS, which experienced a conspicuous damage to the crown (in some trees the defoliation was equal to 90%) showed a sharp decrease in growth. Further, the ability of *P. pinaster* to survive, although the presence of a large fire scar and a possible severe defoliation, is demonstrated by the individual sampled in the MSS presenting the 1993 fire scar. Dendrochronological analyses of that individual highlighted that the plant survived to the 1993 wildfire growing until nowadays. According to the tree-ring dating, that individual, similarly to the rest of the pines in the stand, in 1993 was more than 10 years old with a bark certainly greater than 1 cm. Ryan et al. (1994) applying a prescribed fire on *P. pinaster* stand showed that, with a bark greater than 1 cm of thickness, a scar cannot represent a significant contribution to the tree mortality.

The reduction in growth recorded in 2017, in comparison to the 2016, was evident also in the individuals sampled in the CS and this could be due to the extreme high temperatures recorded in 2017. Indeed, this year has been considered as an extreme dry year (World Meteorological Organization, 2018).

The climate- growth correlations reported in Table 3 showed the importance of temperature on the tree ring width for the whole period. The high temperatures can cause a change in the constant kinetics of the RuBisCo, leading to a consequent decrease in carboxylation rate (Medlyn et al., 2002) and thus to a reduction in tree growth. On the other hand, also precipitation affected the growth of the sampled trees: indeed, in all the four chronologies a reduction in growth has been found after particular dry years: for example, the low rainfall in 1999 (455 mm/year) determined a drastic decline in growth in 2000 in all the trees.

In addition to the climatic factors, competition can also play an important role in the growth rate: dendrochronological analyzes have highlighted how the prescribed burning applications, carried out in 2014 and 2016 in the LSS, have led to a sharp increase in the growth of maritime pine. Prescribed burning, in addition to reducing in competition, through the biomass reduction of the herbaceous and shrubby species, has determined a large nutrients mineralization, ensuring to the dominant individuals to take advantage of the favorable conditions. Similar results were also observed in other prescribed burning studies performed on individuals of *Pinus halepensis* (Battipaglia et al., 2014b; Valor et al., 2018) and *P. pinea* (Battipaglia et al., 2016).

### Ecophysiological Responses of *P. pinaster* to Fire

The isotopic analyses related to 2017 of the four sampled sites allowed to better understand the different processes triggering trees responses to fire. Indeed, the growth reduction found in all the trees was due to changes in ecophysiological mechanisms, mainly related to fire severity. Trees of HSS and MSS sites showed in 2017 a significant increase of δ^18^O and a slight increase of δ^13^C especially of late-wood, compared to previous years. The increase in the oxygen isotopic ratio may be associated with a decrease in stomatal activity, whilst the increase of δ^13^C can indicate a lower partial pressure of CO_2_ in the intracellular spaces of the leaf, due to both photosynthetic activity and stomatal conductance. Therefore, considering both isotopes, according to the dual-isotope approach (Scheidegger et al., 2000) we could hypothesize that the plants to protect themselves from strong stress condition, due to fire, and as a consequences of the serious observed crown damage, closed their stomata and lowered their photosynthetic activity. These conditions, added to the high temperatures and drought conditions through the growing season, explain the observed reduction in growth of 2017 tree ring. Those results are in agreement with the studies carried out by Battipaglia et al. (2014b) on of *Pinus halepensis*, in several forest fires in Southern France, that showed a simultaneous increase in δ^13^C and δ^18^O in occurrence of the fire events. In the same studies the authors demonstrated that the *Pinus halepensis*, considered a species highly vulnerable to fire, was able to survive and to recovery from fire damages, presenting a low mortality rate. To better understand the probability of death of maritime pine, exposed to high severity fire, the study carried out on the individual with the deep fire scar, was crucial. The study showed that, in the periods before the 1993 fire, the tree with the fire scar, located in the MSS, was not subjected to stressful climatic conditions since in the period between 1985 and 1992, precipitation and temperatures were rather constant with low anomalies. In the year of fire, the values of δ^13^C and δ^18^O appeared to drastically increase (Figure 7) and, therefore, the eco-physiological responses were similar to that recorded in the HSS individuals after the 2017 fire.

In the following years from 1993 onwards, while the δ^13^C decreased, returning to the average values, the δ^18^O showed a progressive increase until 1995. This combination suggests that, although the stomatal conductance gradually stabilized over time, a severe defoliation of the plants, at least in the first years after fire, resulted in a lower photosynthetic capacity (Battipaglia et al., 2016), leading a lower growth rate (as recorded by the dendrochronological analyzes). After that period, the growth and the ecophysiological processes of that individual returned to the average values.

The individuals sampled at MSS and LSS showed a strong synchronization in the δ^13^C and δ^18^O chronologies along all the time period (*R* = 0.26 and *R* = 0.24, respectively). However, between 2014 and 2016, the application of the prescribed burning in the LSS determined, in the plants of that site, an increase of δ^13^C while the δ^18^O remained unchanged. The variation of the δ^13^C of the plants of the LSS can be connected to an increase of the photosynthetic activity, triggered by prescribed burning. A positive effect of fire was recorded also in the 2007 when a surface fire has affected individuals of the MSS and when a decrease of δ^13^C and δ^18^O was recorded until 2009, indicating a possible favorable effect of reduction in competition for water among survived plants, as found in a study by Beghin et al. (2011). On the other hand the August 2015 fire, which involved the MSS plants, did not result in significant changes of the isotopic response or drastic changes in growth rate. This allows us to hypothesize that the analyzed pinewood was only marginally affected by this event.

Finally, the isotopic analyses of CS trees showed, in 2017, a moderate decrease of δ^13^C and a drastic increase in δ^18^O. This variation, compared to the previous year, can indicate a lower photosynthetic activity and an unchanged stomatal conductance (Scheidegger et al., 2000) probably related to the extreme hot conditions experienced in that summer by all the plants. The climate-growth correlations demonstrated how the minimum temperature of the summer period (*r* = −0.56^∗∗^), and, in particular, of August (*r* = −0.66^∗∗∗^), has a negative influence on the growth of studied trees. The high temperatures, indeed, besides determining a reduction of the chlorophyll pigments and the denaturation of some thioacid proteins, can cause the inhibition of the photosynthetic process (Bussotti et al., 2012). When air temperature exceeds the compensation point (temperature at which the amount of CO_2_ fixed by photosynthesis is equivalent to that released by respiration), the photosynthetic process is not able to replace the carbon used for respiration. This process implies a consumption of carbohydrates reserve that led the plant to a slowdown or a stop of its growth (Di Toppi et al., 2018). Climate conditions could also represent a cumulative factor of stress contributing to a delayed mortality. Catry et al. (2010) assessing the vegetative condition of 1040 burned trees from 11 different species during the first years after the 2003 wildfire, demonstrated how the tree mortality largely increased after the 2005 drought. Their results emphasize the importance of monitoring the tree health in the following years. The burned trees in the study sites showed, at the end of 2018, a very limited mortality rate, with only the 2–10% of plants reducing their vigor. We will continue monitoring the area and the analyzed trees since our work want to be a contribution on the fire ecology of the *P. pinaster species*, underling the importance of evaluating the ecophysiological responses of the species to fire severity in order to valuate the most suitable and effective silvicultural operations mainly in post-fire forest management. Indeed, *P. pinaster* trees affected by fire, with needles scorched or consumed, are not necessarily dead and before to remove every blackened trees, it is important to assess the real damage, waiting for the next growing season, in particular in Natural parks or in areas not close to roads or anthropic settlements. Savage logging could produce severe damage to soil stability and biodiversity, and in particular mechanical actions can largely increase seedling mortality (e.g., Martinez-Sanchez et al., 1995; Fernandes et al., 2008), negatively affecting the ratios final seedling density/initial seedling density and final seedling density/total viable seed dispersal (Vega et al., 2008).

## Author Contributions

GB conceived and designed the study. FN performed sampling and analyses. SA and AE contributed to the analyses. FN and GB wrote the main part of the manuscript. All authors contributed to interpretation of the overall data and wrote specific parts, made a critical revision of the whole text, and approved the submitted version of the manuscript.

## Conflict of Interest Statement

The authors declare that the research was conducted in the absence of any commercial or financial relationships that could be construed as a potential conflict of interest. The handling Editor is currently organizing a Research Topic with one of the authors GB and confirms the absence of any other collaboration.

## Footnotes

1 https://climexp.knmi.nl

## Supplementary Material

FIGURE S1 Trend of total annual precipitation in green (expressed in millimeters/year) and average temperatures in blue (expressed in °C) recorded from 1975 to 2017 and coming from the KNMI Climate Explorer database.

FIGURE S2 Individual of *P. pinaster* belonging to the MSS which has an important lesion on the trunk due to a previous fire, dated 1993.

FIGURE S3 Relationship between ring growth of 2017 and the percentage of crown scorch of the MSS trees (indicated with in yellow symbols) and LSS trees (indicated in red).
