# Supplementary figures and images for: Fire Severity Influences Ecophysiological Responses of Pinus pinaster Ait
Source: Front Plant Sci. 2019 Apr 26;10:539. doi: 10.3389/fpls.2019.00539 (PMC6499007; doi:10.3389/fpls.2019.00539)

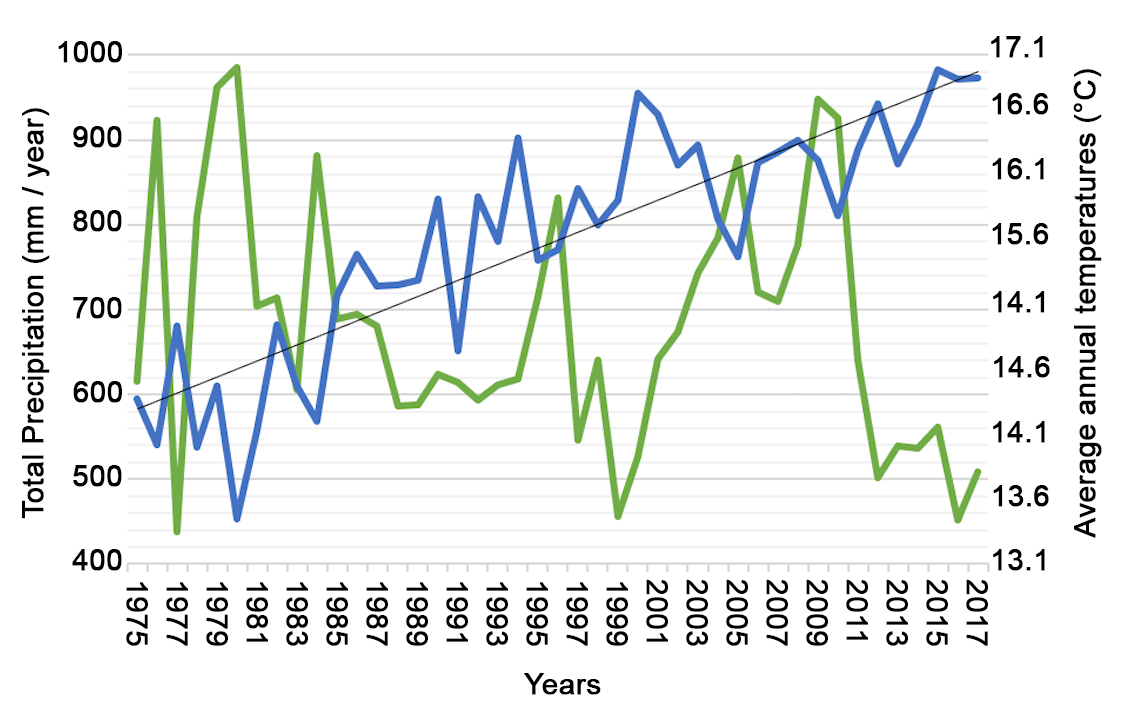

Supplement: FIGURE S1 — Trend of total annual precipitation in green (expressed in millimeters/year) and average temperatures in blue (expressed in °C) recorded from 1975 to 2017 and coming from the KNMI Climate Explorer database. [file Image_1.JPEG]

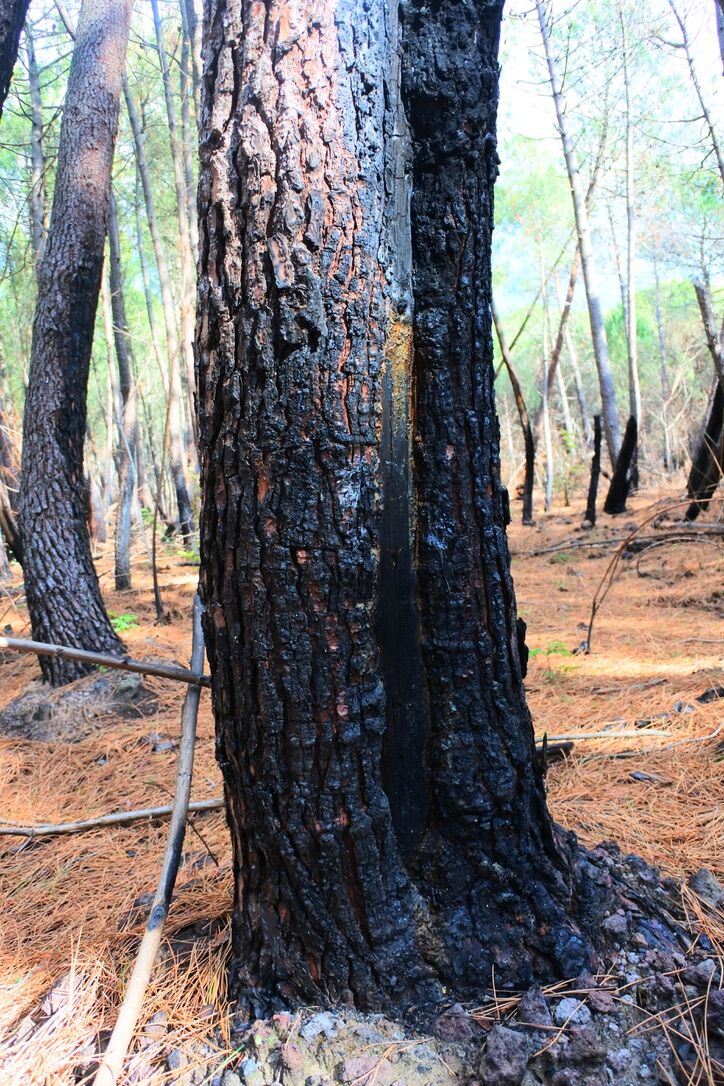

Supplement: FIGURE S2 — Individual of P. pinaster belonging to the MSS which has an important lesion on the trunk due to a previous fire, dated 1993. [file Image_2.JPEG]

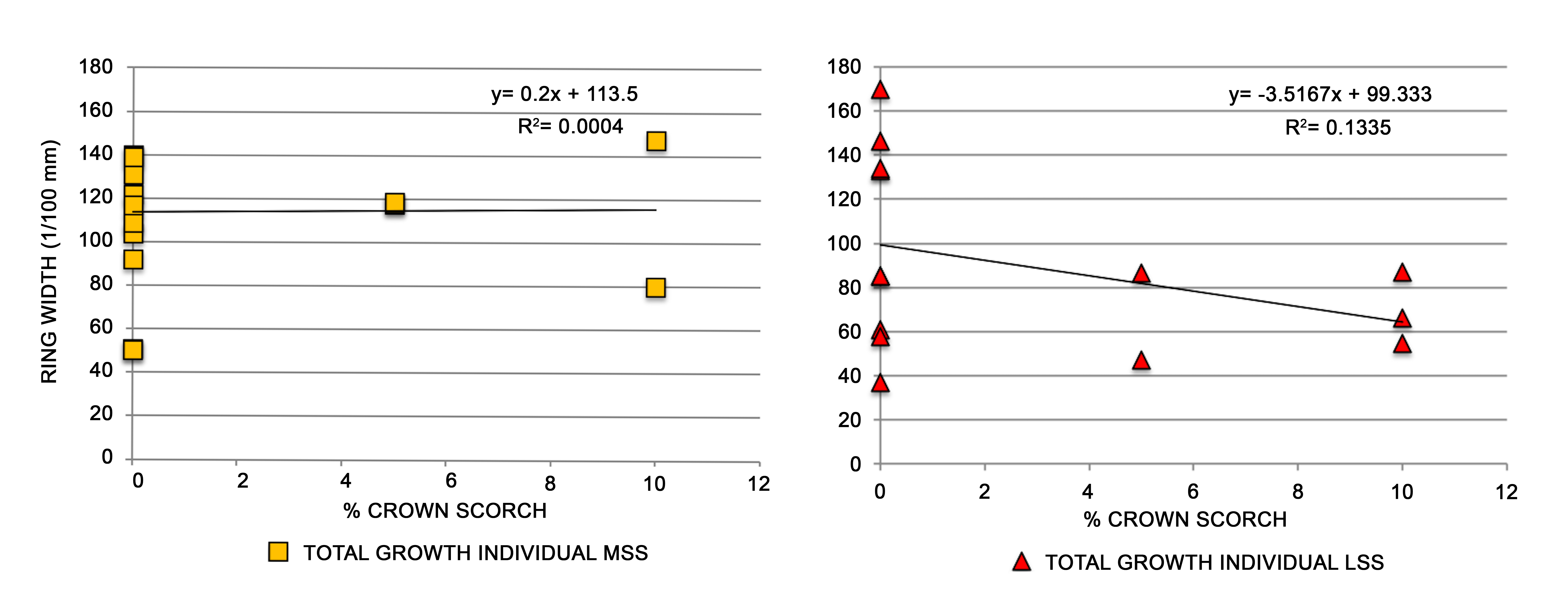

Supplement: FIGURE S3 — Relationship between ring growth of 2017 and the percentage of crown scorch of the MSS trees (indicated with in yellow symbols) and LSS trees (indicated in red). [file Image_3.JPEG]
